# Synthesis and Anti-Breast Cancer Evaluation of Novel *N*-(Guanidinyl)benzenesulfonamides

**DOI:** 10.3390/ijms15045582

**Published:** 2014-04-01

**Authors:** Mostafa M. Ghorab, Marwa G. El-Gazzar, Mansour S. Alsaid

**Affiliations:** 1 Department of Pharmacognosy, College of Pharmacy, King Saud University, P.O. Box 2457, Riyadh 11451, Saudi Arabia; E-Mail: msalsaid@ksu.edu.sa; 2 Department of Drug Radiation Research, National Center for Radiation Research and Technology, Nasr City, Cairo 11371, Egypt; E-Mail: marwagalalgazzar@yahoo.com

**Keywords:** sulfonamides, heterocycles, structure-activity relationships, antitumor agents

## Abstract

A series of 4-(substituted)-*N*-(guanidinyl)benzenesulfonamides bearing biologically active pyrazole, pyrimidine and pyridine moieties were prepared and evaluated for their anticancer activity against human tumor breast cell line (MCF7). These sulfonamides showed promising activity with *IC*_50_ values ranging from 49.5 to 70.2 μM. The structure-activity relationship of the synthesized compounds was studied. Interestingly, it was found that the most potent compounds in this study were the corresponding 2-cyanoacrylate **3**, 3-oxobutanoate **4**, pyrazole **6**, pyridine **9** and pyrazole **13**. Compounds **7** and **8** are nearly as active as Doxorubicin as reference drug with (*IC*_50_ values = 70.2, 68.1 μM), while compounds **5**, **10** and **11** exhibited a moderate activity.

## 1. Introduction

Cancer, known medically as malignant neoplasia, is a broad group of diseases involving unregulated cell growth. In cancer, cells divide and grow uncontrollably, forming malignant tumors, which may invade nearby parts of the body. The cancer may also spread to more distant parts of the body through the lymphatic system or bloodstream [1,2]. Breast cancer is a kind of cancer that develops from breast cells. Breast cancer usually starts off in the inner lining of milk ducts or the lobules that supply them with milk. A malignant tumor can spread to other parts of the body [3,4]. The vast majority of breast cancer cases occur in females and breast cancer is the most common invasive cancer in females worldwide. It accounts for 16% of all female cancers and 22.9% of invasive cancers in women. Including both males and females, 18.2% of all cancer deaths worldwide are from breast cancer [5,6]. Treatments for breast cancer include surgery as well as radiation, chemotherapy and hormonal therapy; these treatments are either local or systemic. Local treatments, such as surgery and radiation therapy, remove, destroy or control cancer cells in specific areas. Systemic treatments, such as chemotherapy and hormonal therapy, destroy or control cancer throughout the body. Depending on the condition, one may receive one treatment or a combination at the same time or in succession [7].

**Figure 1.**
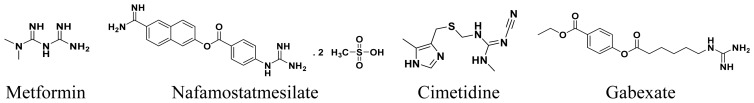
Some commercially available guanidine containing drugs.

Nitrogen heterocycles are of special interest because they constitute an important class of natural and non-natural products, many of which exhibit useful biological activities. Pyrazoles and their derivatives exhibit a broad spectrum of biological activities such as antimicrobial [8], anti-inflammatory [9], and antitumor activities [10]. With growing application on their synthesis and bioactivity, chemists and biologists in recent years have directed considerable attention on the research of pyrazole derivatives. Pyrimidines are of chemical and pharmacological interest and compounds containing the pyrimidine ring system have been shown to possess antimicrobial [11,12], antimalarial [13], anticonvulsant [14], and antitumor activities [15]. Some are valuable drugs for the treatment of acute leukemia in children and adult granulocytic leukemia [16]. Compounds containing a pyridine moiety as a precursor have proven to possess several biologically properties especially as anticancer agents [17–19]. These findings led us to enlarge our investigations and to continue working on the synthesis of biologically active compounds [20–25] to synthesize a series of 4-(substituted)-*N*-(guanidinyl)benzenesulfonamides in order to evaluate if the presence of the guanidine moiety could enhance their biological activities. The choice of guanidine derivatives is due to its presence in several classes of pharmacological active compounds, recently anticancer drugs [26,27], and also in some commercially available drugs for example, the anti-diabetic metformin which is an oral anti-diabetic drug in the biguanide class and the first-line drug of choice for the treatment of type 2 diabetes [28–30]; the anticoagulant Nafamostatmesilate [31]; cimetidine which is a histamine H2-receptor antagonist that inhibits stomach acid production and largely used in the treatment of heartburn and peptic ulcers [32]. Gabexate is a serine protease inhibitor which is used therapeutically (as Gabexatemesilate) in the treatment of pancreatitis, and disseminated intravascular coagulation [33] (Figure 1).

## 2. Results and Discussion

### 2.1. Chemistry

**Scheme 1.**
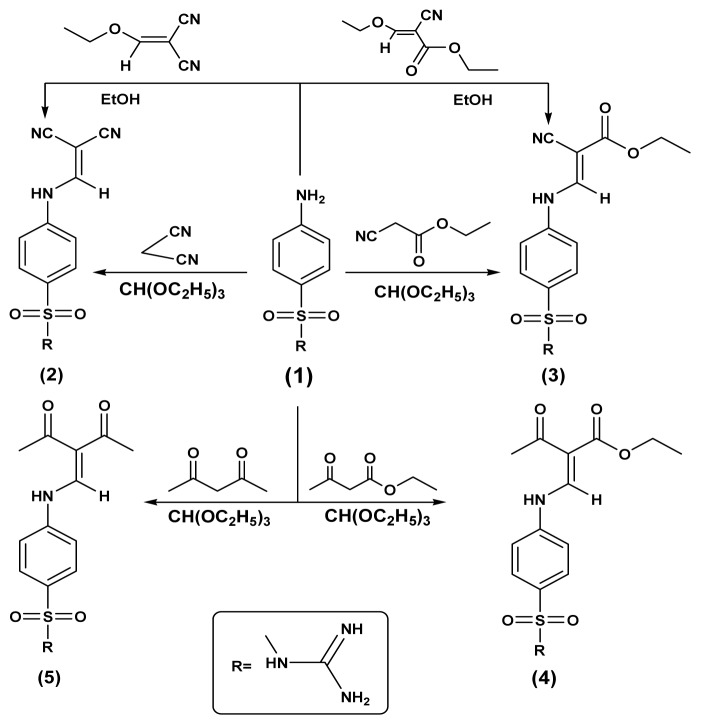
Synthetic pathways for compounds **2**–**5**.

**Scheme 2.**
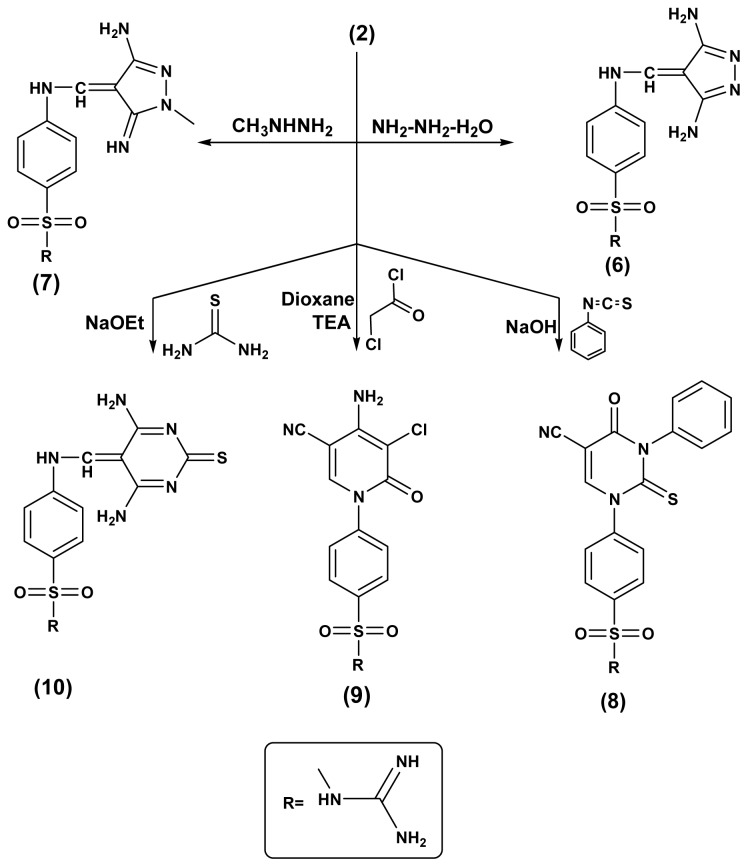
Synthetic pathways for compounds **6**–**10**.

**Scheme 3.**
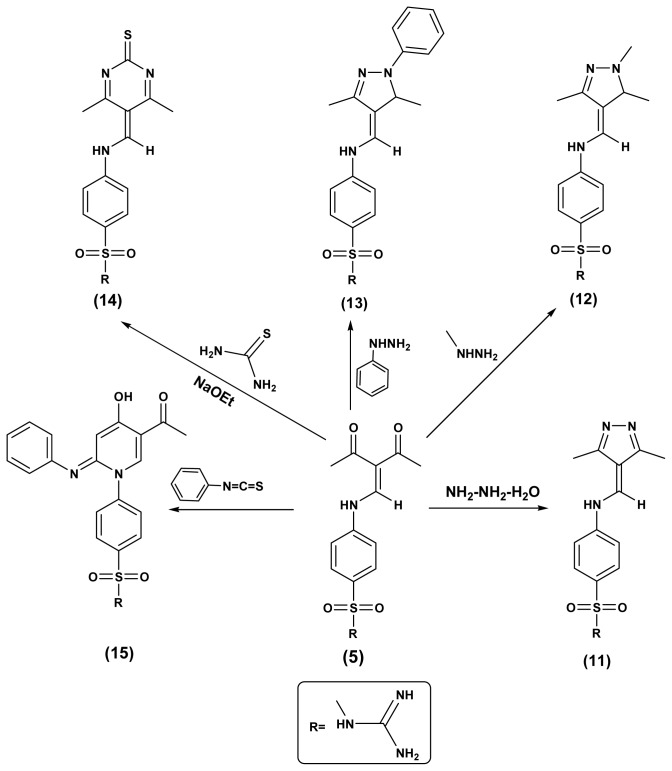
Synthetic pathways for compounds **11**–**15**.

In this work, the reactivity of sulfaguanidine1 with active methylenes (malononitril, 2-(ethoxymethylene)malononitrile, ethylcyanoacetate, (ethoxymethylene)ethylcyanoacetate, ethylacetoacetate and acetylacetone) was studied. The reactions proceeded according to the reported method [24] and the obtained sulfonamide derivatives **2**–**5**, respectively, were identified by elemental and spectral data (Scheme 1). Due to the biological importance of pyrazole, pyridine and pyrimidine rings as anticancer agents, the strategic starting materials sulfonamide derivatives **2** and **5** were reacted with different nucleophiles in order to obtain biologically active pyrazole, pyridine and pyrimidine derivatives bearing sulfonamide moieties. Thus, interaction of sulfonamide derivatives **2** with either hydrazine hydrate or methylhydrazine yielded the corresponding pyrazole derivatives **6** and **7**, the formation of the pyrazole ring was proved by the disappearance of CN in the infrared spectra and the appearance of NH_2_ bands which is also traced in their ^1^H-NMR spectra and were exchangeable with D_2_O, ^1^H-NMR spectrum of compound **7** showed a singlet at 2.4 ppm corresponding to the methyl group, while, their ^13^C-NMR spectra showed the appearance of signals for the carbons of the pyrazole rind at 166.4 ppm for compound **6** and 163.8 ppm for compound **7**. On the other hand, interaction of compound **2** with chloroacetyl chloride in dioxane as a solvent containing triethylamine as catalyst yielded the corresponding pyridine derivatives **9** which was characterized by the presence of CN band and appearance of new bands for NH_2_, carbonyl and C-Clgroups in the infrared spectrum and the specific signals of the pyridine ring appeared in the ^13^C-NMR spectrum. Reaction of compound **2** with phenyl isothiocyanate in NaOH/ethanol and/or thiourea gave the corresponding pyrimidine-2-thione derivatives **8** or **10**, respectively and their structure were proved by microanalytical and spectral data (Scheme 2). Similarily, the sulfonamide derivative **5** was reacted with either hydrazine hydrate, methylhydrazine or phenylhydrazine yielded the corresponding pyrazole derivatives **11**–**13**, respectively. On the other hand, interaction of compound **5** with thiourea and/or phenyl isothiocyanate in NaOH/ethanol gave the corresponding pyrimidine and pyridine derivatives **14** and **15**, respectively (Scheme 3).

### 2.2. In-Vitro Anticancer Screening

Doxorubicin, the reference drug used in this study is a drug used in cancer chemotherapy. It is an anthracycline antibiotic, it works by intercalating DNA and inhibition of macromolecular biosynthesis. This inhibits the progression of the enzyme topoisomerase II, which relaxes supercoils in DNA for transcription. Doxorubicin stabilizes the topoisomerase II complex after it has broken the DNA chain for replication, preventing the DNA double helix from being resealed and thereby stopping the process of replication. It is commonly used in the treatment of a wide range of cancers as in acute leukemia’s Hodgkin’s disease, and other lymphomas and cancers of the breast, adrenal cortex, endometrium, lung, ovary, and other sites [34]. The relationship between surviving fraction and drug concentration was plotted; the response parameters calculated was *IC*_50_ value, which corresponds to the compound concentration that causes 50% inhibition of cellular viability (Table 1). From Table 1, we can observe that all the tested compounds were found to be equipotent or even more potent than Doxorubicin on the MCF7 cell line with *IC*_50_ values ranging from 49.5 to 70.2 μM. The most potent compounds in this study are compound **3** bearing 2-cyanoacrylate moiety (*IC*_50_ = 49.5 μM), 3-oxobutanoate derivative **4** (*IC*_50_ = 54.8 μM), pyrazole derivative **6** (*IC*_50_ = 59.0 μM), pyridine **9** (*IC*_50_ = 59.2 μM) and the pyrazole derivative **13** (*IC*_50_ = 57.8 μM), and they were found to be more potent than the reference drug Doxorubicin (*IC*_50_ = 71.8 μM). On the other hand, the pyrazole derivative **7** and pyrimidine **8** were found to be nearly as potent as the positive control Doxorubicin with *IC*_50_ = 70.2, 68.1 μM, respectively. In addition compounds **5**, **10**, and **11** exhibited a moderate activity with *IC*_50_ = 75.3, 76.1, 75.6 μM, respectively. Finally, the least potent are compounds **12** (*IC*_50_ = 102.9 μM) and **14**, which showed no activity on MCF7 cell line. These results attract the attention of the possible use of the synthesized pyrazole, pyridine and pyrimidine derivatives carrying a biologically active sulfonamide moiety for treatment of breast tumors and these results are in agreement with previous studies done on pyrazole, pyridine and pyrimidine and other sulfonamide derivatives and resulted in compounds with promising anticancer activities [20–25].

## 3. Experimental Section

### 3.1. Chemistry

Reagents were obtained from commercial suppliers and were used without purification. Melting points were determined in open capillary tubes using Thermo system FP800 Mettler FP80 central processor (Stuart Scientific, Redhill, UK) supplied with FP81 MBC cell apparatus, and were uncorrected. Elemental analyses (C, H, N) were performed on a Perkin-Elmer 2400 Instrument (Perkin-Elmer, Norwalk, CT, USA). All compounds were within ±0.4% of the theoretical values. Infrared (IR) spectra (KBr disc) were recorded on FT-IR spectrophotometer (Perkin Elmer, Norwalk, CT, USA) at the Research Center, College of Pharmacy, King Saud University, Saudi Arabia.^1^H- and ^13^C-NMR spectra were recorded on a Ultra Shield Plus 500 MHz (Bruker, Munich, Germany) spectrometer operating at 500 MHz for proton and 125 MHz for carbon, respectively. The chemical shift values are reported in δ (ppm) relative to the residual solvent peak, the coupling constants (*J*) are reported in Hertz (Hz).

#### 3.1.1. *N*-Carbamimidoyl-4-(2,2-cyanovinylamino)benzenesulfonamide (**2**)

A mixture of sulfaguanidine **1** (2.14 g, 0.01 mol), malononitrile (0.66 g, 0.01 mol), triethylorthoformate (1.48 g, 0.01 mol), and acetic acid (1 mL) in methanol (30 mL) was refluxed for 5 h, the reaction mixture was filtered, the filtered solid was crystallized from ethanol to give **2**. Yield%: 85, m.p. = 274.6 °C, IR, cm^−1^: 3446, 3338, 3205 (NH, NH_2_), 3100 (CH arom.), 2930, 2846 (CH aliph.), 2212 (CN), 1634 (C=N), 1368, 1151 (SO_2_). ^1^H-NMR (DMSO-*d*_6_, ppm): 6.1[s, 1H, CH], 6.7[s, 2H, NH_2_, D_2_O-exchangeable], 7.5, 7.7[2d, 4H, Ar–H, AB system, *J =* 7.1 Hz], 8.5[s, 1H, NH–imino, D_2_O-exchangeable], 11.3[s, 2H, NH + SO_2_NH, D_2_O-exchangeable]. ^13^C-NMR (DMSO-*d*_6_, ppm): 56.0, 113.8 (2), 117.7 (2), 126.0 (2), 140.6, 141.2, 155.8, 158.0. Anal. Calcd for C_11_H_10_N_6_O_2_S (290): C, 45.51; H, 3.47; N, 28.95. Found: C, 45.21; H, 3.80; N, 28.62.

#### 3.1.2. (*E*)-Ethyl-3-(4-(*N*-carbamimidoylsulfamoyl)phenylamino)-2-cyanoacrylate (**3**)

A mixture of sulfaguanidine **1** (2.14 g, 0.01 mol), ethylcyanoacetate (1.13 g, 0.01 mol), triethylorthoformate (1.48 g, 0.01 mol), and acetic acid (1 mL) in methanol (30 mL) was refluxed for 5 h, the reaction mixture was filtered, the filtered solid was crystallized from ethanol to give **3**. Yield%: 83, m.p. = 143.6 °C, IR, cm^−1^: 3433, 3381, 3291 (NH, NH_2_), 3056 (CH arom.), 2961, 2836 (CH aliph.), 2220 (CN), 1713 (C=O), 1635 (C=N), 1362, 1168 (SO_2_). ^1^H-NMR (DMSO-*d*_6_, ppm): 1.2[t, 3H, CH_3_], 4.1[q, 2H, CH_2_], 5.7[s, 2H, NH_2_, D_2_O-exchangeable], 6.5–7.7[m, 5H, Ar–H + CH], 8.5[s, 1H, NH–imino, D_2_O-exchangeable], 11.0[s, 2H, NH + SO_2_NH, D_2_O-exchangeable]. ^13^C-NMR (DMSO-*d*_6_, ppm): 13.8, 61.9, 76.1, 112.1, 117.6 (2), 127.2 (2), 130.6, 141.8, 151.3, 158.0, 164.2. Anal. Calcd for C_13_H_15_N_5_O_4_S (337): C, 46.28; H, 4.48; N, 20.76. Found: C, 46.51; H, 4.80; N, 20.44.

#### 3.1.3. (*E*)-Ethyl-2-((4-(*N*-carbamimidoylsulfamoyl)phenylamino)methylene)-3-oxobutanoate (**4**)

A mixture of sulfaguanidine **1** (2.14 g, 0.01 mol), ethylacetoacetate (1.30 g, 0.01 mol), triethylorthoformate (1.48 g, 0.01 mol), and acetic acid (1 mL) in methanol (30 mL) was refluxed for 5 h, the reaction mixture was filtered, the filtered solid was crystallized from ethanol to give **4**. Yield%: 83, m.p. = 173.9 °C, IR, cm^−1^: 3433, 3390, 3236 (NH, NH_2_), 3100 (CH arom.), 2919, 2880 (CH aliph.), 1710, 1699 (2C=O), 1612 (C=N), 1384, 1167 (SO_2_). ^1^H-NMR (DMSO-*d*_6_, ppm): 1.2[t, 3H, CH_3_], 2.3[s, 3H, COCH_3_], 4.1[q, 2H, CH_2_], 5.6[s, 2H, NH_2_, D_2_O-exchangeable], 6.5, 7.4[2d, 4H, Ar–H, AB system, *J =* 7.3 Hz], 8.4[s, 1H, NH–imino, D_2_O-exchangeable], 10.2[s, 2H, NH, SO_2_NH, D_2_O-exchangeable]. ^13^C-NMR (DMSO-*d*_6_, ppm): 13.9, 24.8, 59.6, 102.7, 118.2 (2), 126.5 (2), 127.2, 148.8, 151.3, 157.7, 169.1, 194.9. Anal. Calcd for C_14_H_18_N_4_O_5_S (354): C, 47.45; H, 5.12; N, 15.81. Found: C, 47.12; H, 4.80; N, 15.44.

#### 3.1.4. 4-(2-Acetyl-3-oxobut-1-enylamino)-*N*-carbamimidoylbenzenesulfonamide (**5**)

A mixture of sulfaguanidine **1** (2.14 g, 0.01 mol), acetylacetone (1.00 g, 0.01 mol), triethylorthoformate (1.48 g, 0.01 mol), and acetic acid (1 mL) in methanol (30 mL) was refluxed for 5 h, the reaction mixture was filtered, the filtered solid was crystallized from ethanol to give **5**. Yield%: 81, m.p. = 162.0 °C, IR, cm^−1^: 3435, 3339, 3132 (NH, NH_2_), 3099 (CH arom.), 2961, 2834 (CH aliph.), 1721, 1701 (2C=O), 1612 (C=N), 1384, 1156 (SO_2_). ^1^H-NMR (DMSO-*d*_6_, ppm): 2.4[s, 6H, 2COCH_3_], 6.6[s, 2H, NH_2_, D_2_O-exchangeable], 7.3, 7.7[2d, 4H, Ar–H, AB system, *J =* 7.0 Hz], 7.9[s, 1H, CH], 8.8[s, 1H, NH–imino, D_2_O-exchangeable], 12.5[s, 2H, NH, SO_2_NH, D_2_O-exchangeable]. ^13^C-NMR (DMSO-*d*_6_, ppm): 23.0 (2), 112.3, 118.1 (2), 127.1 (2), 129.4, 140.2, 142.2, 158.9, 196.1 (2). Anal. Calcd for C_13_H_16_N_4_O_4_S (324): C, 48.14; H, 4.97; N, 17.27. Found: C, 48.34; H, 4.80; N, 17.47.

#### 3.1.5. *N*-Carbamimidoyl-4-((3,5-diamino-4*H*-pyrazol-4-ylidene)methylamino)benzensulfonamide (**6**)

A mixture of **2** (2.90 g, 0.01 mol) and hydrazine hydrate (0.50 g, 0.01 mol) in dioxane (20 mL) was refluxed for 5 h, the reaction mixture was cooled, poured onto ice water. The precipitated solid product was filtered and crystallized from methanol to give compound **6**. Yield%: 76, m.p. = 117.6 °C, IR, cm^−1^: 3398, 3340, 3276 (NH, NH_2_), 3066 (CH arom.), 2961, 2836 (CH aliph.), 1618 (C=N), 1384, 1179 (SO_2_). ^1^H-NMR (DMSO-*d*_6_, ppm): 6.54[s, 2H, NH_2_, D_2_O-exchangeable], 6.59[s, 4H, 2NH_2_-pyrazole, D_2_O-exchangeable], 6.9–7.4[m, 5H, Ar–H + CH], 8.4[s, 1H, NH–imino, D_2_O-exchangeable], 9.4[s, 2H, NH, SO_2_NH, D_2_O-exchangeable]. ^13^C-NMR (DMSO-*d*_6_, ppm): 112.3, 115.3 (2), 127.2 (2), 130.7, 144.0, 151.3, 157.7, 166.4 (2). Anal. Calcd for C_11_H_14_N_8_O_2_S (322): C, 40.99; H, 4.38; N, 34.76. Found: C, 40.76; H, 4.67; N, 34.45.

#### 3.1.6. 4-((3-Amino-5-imino-1-methyl-1*H*-pyrazol-4-(5*H*)-ylidene)methylamino)-*N*-carbamimidoyl-benzenesulfonamide (**7**)

A mixture of **2** (2.90 g, 0.01 mol) and methylhydrazine (0.46 g, 0.01 mol) in dioxane (20 mL) was refluxed for 5 h, the reaction mixture was cooled, poured onto ice water. The precipitated solid products were filtered and crystallized from methanol to give compound **7**. Yield%: 89, m.p. = 129.4 °C, IR, cm^−1^: 3401, 3378, 3312 (NH, NH_2_), 3026 (CH arom.), 2981, 2860 (CH aliph.), 1595 (C=N), 1368, 1156 (SO_2_). ^1^H-NMR (DMSO-*d*_6_, ppm): 2.4[s, 3H, CH_3_], 6.54[s, 2H, NH_2_, D_2_O-exchangeable], 6.56[s, 2H, NH_2_pyrazole, D_2_O-exchangeable], 7.3–7.5[m, 6H, Ar–H + CH + NH pyrazole], 7.7[s, 1H, NH–imino, D_2_O-exchangeable], 9.3[s, 2H, NH + SO_2_NH, D_2_O-exchangeable]. ^13^C-NMR (DMSO-*d*_6_, ppm): 34.5, 112.3, 115.2 (2), 127.2 (2), 130.7, 139.7, 151.3, 151.4, 157.7, 163.8. Anal. Calcd for C_12_H_16_N_8_O_2_S (336): C, 42.85; H, 4.79; N, 33.31. Found: C, 42.56; H, 4.98; N, 33.67.

#### 3.1.7. *N*-Carbamimidoyl-4-(5-cyano-4-oxo-3-phenyl-2-thioxo-3,4-dihydropyrimidin-1(2*H*)-yl)-benzenesulfonamide (**8**)

A mixture of **2** (2.90 g, 0.01.mol), phenyl isothiocyanate (1.35 g, 0.01 mol) and sodium hydroxide (0.40 g, 0.01 mol) in ethanol (20 mL) was refluxed for 3 h. The reaction mixture was cooled, poured onto ice water, acidified with dilute HCl, then the solid product was filtered and crystallized from dioxane to give **8**. Yield%: 88, m.p. = 76.4 °C, IR, cm^−1^: 3217, 3186, 3109 (NH, NH_2_), 3030 (CH arom.), 2982, 2861 (CH aliph.), 2193 (CN), 1374, 1199 (SO_2_), 1291 (C=S). ^1^H-NMR (DMSO-*d*_6_, ppm): 6.9[s, 2H, NH_2_, D_2_O-exchangeable], 7.1–7.6[m, 9H, Ar–H], 8.3[s, 1H, CH–pyrimidine], 11.0[s, 2H, NH, SO_2_NH, D_2_O-exchangeable]. ^13^C-NMR (DMSO-*d*_6_, ppm): 94.9, 118.1, 121.6 (2), 122.9, 124.9 (2), 128.4 (2), 128.6 (2), 137.7, 138.5, 139.2, 153.5, 160.6, 168.1, 187.7 (C=S). Anal. Calcd for C_18_H_14_N_6_O_3_S_2_ (426): C, 50.69; H, 3.31; N, 19.71. Found: C, 50.35; H, 3.66; N, 19.51.

#### 3.1.8. 4-(4-Amino-3-chloro-5-cyano-2-oxopyridin-1(2*H*)-yl)-*N*-carbamimidoylbenzenesulfonamide (**9**)

A mixture of **2** (2.90 g, 0.01.mol) and chloroacetyl chloride (1.13 g, 0.01 mol) in dioxane (20 mL) containing 3 drops of triethylamine was refluxed for 3 h. The reaction mixture was cooled, poured onto ice water and the solid product was filtered and crystallized from ethanol to give **9**. Yield%: 79, m.p. = 211.4 °C, IR, cm^−1^: 3408, 3276, 3204 (NH, NH_2_), 3100 (CH arom.), 2226 (CN), 1736 (C=O), 1326, 1189 (SO_2_), 829 (C–Cl). ^1^H-NMR (DMSO-*d*_6_, ppm): 4.4[s, 2H, NH_2_–pyridine, D_2_O-exchangeable], 7.5[s, 2H, NH_2_, D_2_O-exchangeable], 7.6–8.0[m, 4H, Ar–H], 8.6[s, 1H, CH–pyridine], 11.3[s, 2H, NH, SO_2_NH, D_2_O-exchangeable]. ^13^C-NMR (DMSO-*d*_6_, ppm): 91.6, 98.3, 115.9, 118.9 (2), 127.4 (2), 136.6, 138.3, 146.2, 155.7, 166.3, 169.6. Anal. Calcd for C_13_H_11_ClN_6_O_3_S (366): C, 42.57; H, 3.02; N, 22.91. Found: C, 42.31; H, 3.36; N, 22.67.

#### 3.1.9. *N*-Carbamimidoyl-4-((4,6-diamino-2-thioxopyrimidin-5(2*H*)-ylidene)methylamino)-benzenesul-fonamide (**10**)

A mixture of **2** (2.90 g, 0.01 mol) and thiourea (0.76 g, 0.01 mol) was refluxed for 5 h in ethanol (20 mL) containing sodium ethoxide (0.01 mol). The reaction mixture was cooled, poured onto ice water, acidified with dilute HCl, then the precipitated solid product was filtered and crystallized from methanol to give **10**. Yield%: 90, m.p. > 350 °C, IR, cm^−1^: 3434, 3391, 3309 (NH, NH_2_), 3088 (CH arom.), 2976, 2819 (CH aliph.), 1384, 1151 (SO_2_), 1229 (C=S). ^1^H-NMR (DMSO-*d*_6_, ppm): 6.4[s, 2H, NH_2_, D_2_O-exchangeable], 6.9[s, 4H, 2NH_2_–pyrimidine, D_2_O-exchangeable], 7.9–8.3[m, 5H, Ar–H + CH], 10.3[s, 1H, NH, D_2_O-exchangeable], 11.7[s, 2H, NH, SO_2_NH, D_2_O-exchangeable]. ^13^C-NMR (DMSO-*d*_6_, ppm): 94.1, 116.5 (2), 127.7 (2), 129.3, 138.2, 143.8, 161.8, 163.6 (2), 218.9. Anal. Calcd for C_12_H_14_N_8_O_2_S_2_ (366): C, 39.33; H, 3.85; N, 30.58. Found: C, 39.62; H, 3.59; N, 30.23.

#### 3.1.10. *N*-Carbamimidoyl-4-((3,5-dimethyl-4*H*-pyrazol-4-ylidene)methylamino)benzensulfonamide (**11**)

Compound **5** (3.24 g, 0.01 mol) was mixed with hydrazine hydrate (0.50 g, 0.01 mol) in dioxane (20 mL) and refluxed for 5 h, the reaction mixture was cooled, poured onto ice water. The precipitated solid products were filtered and crystallized from methanol to give compound **11**. Yield%: 90, m.p. = 113.9 °C, IR, cm^−1^: 3399, 3368, 3204 (NH, NH_2_), 3039 (CH arom.), 2944, 2879 (CH aliph.), 1617 (C=N), 1308, 1179 (SO_2_). ^1^H-NMR (DMSO-*d*_6_, ppm): 2.1[s, 6H, 2CH_3_], 6.6[s, 2H, NH_2_, D_2_O-exchangeable], 7.3–7.6[m, 5H, Ar–H + CH], 9.3[s, 1H, NH, D_2_O-exchangeable], 11.1[s, 2H, NH–imino + SO_2_NH, D_2_O-exchangeable]. ^13^C-NMR (DMSO-*d*_6_, ppm): 23.0 (2), 103.1, 112.3 (2), 127.2 (2), 130.2, 130.7, 151.3, 157.7, 167.2 (2). Anal. Calcd for C_13_H_16_N_6_O_2_S (320): C, 48.74; H, 5.03; N, 26.23. Found: C, 48.99; H, 5.41; N, 26.01.

#### 3.1.11. *N*-Carbamimidoyl-4-((1,3,5-trimethyl-1*H*-pyrazol-4-yl)methyleneamino)benzenesulfonamide (**12**)

Compound **5** (3.24 g, 0.01 mol) was mixed with methylhydrazine (0.4 g, 0.01 mol) in dioxane (20 mL) and refluxed for 5 h, the reaction mixture was cooled, poured onto ice water. The precipitated solid product was filtered and crystallized from methanol to give compound **12**. Yield%: 76, m.p. = 171.8 °C, IR, cm^−1^: 3394, 3351, 3391 (NH, NH_2_), 3100 (CH arom.), 2976, 2852 (CH aliph.), 1620 (C=N), 1309, 1178 (SO_2_). ^1^H-NMR (DMSO-*d*_6_, ppm): 2.5[s, 6H, 2CH_3_], 3.4[s, 3H, NCH_3_], 5.6[s, 2H, NH_2_, D_2_O-exchangeable], 6.5–7.3[m, 4H, Ar–H + CH], 7.4[s, 1H, NH–imino, D_2_O-exchangeable], 8.9[s, 2H, NH + SO_2_NH, D_2_O-exchangeable]. ^13^C-NMR (DMSO-*d*_6_, ppm): 13.2, 17.3, 40.0, 112.3, 125.4 (2), 127.2 (2), 136.7, 146.8, 151.3, 157.7, 161.4, 177.8. Anal. Calcd for C_14_H_18_N_6_O_2_S (334): C, 50.28; H, 5.43; N, 25.13. Found: C, 50.67; H, 5.08; N, 25.36.

#### 3.1.12. *N*-Carbamimidoyl-4-((3,5-dimethyl-1-phenyl-1*H*-pyrazol-4-yl)methyleneamino)-benzenesulf-onamide (**13**)

Compound **5** (3.24 g, 0.01 mol) was mixed with phenylhydrazine (1.08 g, 0.01 mol) in dioxane (20 mL) and refluxed for 5 h, the reaction mixture was cooled, poured onto ice water. The precipitated solid product was filtered and crystallized from methanol to give compound **13**. Yield%: 87, m.p. = 100.8 °C, IR, cm^−1^: 3386, 3320, 3220 (NH, NH_2_), 3099 (CH arom.), 2955, 2836 (CH aliph.), 1598 (C=N), 1384, 1130 (SO_2_). ^1^H-NMR (DMSO-*d*_6_, ppm): 2.5[s, 6H, 2CH_3_], 6.0[s, 2H, NH_2_, D_2_O-exchangeable], 6.5–7.7[m, 9H, Ar–H^+^], 9.7[s, 1H, NH–imino, D_2_O-exchangeable], 9.9[s, 2H, NH + SO_2_NH, D_2_O-exchangeable]. ^13^C-NMR (DMSO-*d*_6_, ppm): 12.1 (CH_3_), 13.2 (CH_3_), 117.3, 121.3 (2), 124.0 (2), 126.6, 127.3 (2), 128.6 (2), 139.0, 139.6, 147.4, 150.8, 157.6, 158.0, 172.1. Anal. Calcd for C_19_H_20_N_6_O_2_S (396): C, 57.56; H, 5.08; N, 21.20. Found: C, 57.36; H, 5.24; N, 21.59.

#### 3.1.13. *N*-Carbamimidoyl-4-((4,6-dimethyl-2-thioxopyrimidin-5(2*H*)-ylidene)methylamino) benzene-sulfonamide (**14**)

A mixture of **5** (3.24 g, 0.01 mol) and thiourea (0.76 g, 0.01 mol) was refluxed for 5 h in ethanol (20 mL) containing sodium ethoxide (0.01 mol). The reaction mixture was cooled, poured onto ice water, acidified with dilute HCl, then the precipitated solid product was filtered and crystallized from methanol to give **14**. Yield%: 89, m.p. > 350 °C, IR, cm^−1^: 3427, 3393, 3286 (NH, NH_2_), 3076 (CH arom.), 2971, 2876 (CH aliph.), 1636 (C=N), 1386, 1161 (SO_2_), 1246 (C=S). ^1^H-NMR (DMSO-*d*_6_, ppm): 0.8[s, 6H, 2CH_3_], 6.4[s, 2H, NH_2_, D_2_O-exchangeable], 7.2–7.4[m, 5H, Ar–H + CH], 8.7[s, 1H, NH–imino, D_2_O-exchangeable], 9.4[s, 2H, NH, SO_2_NH, D_2_O-exchangeable]. ^13^C-NMR (DMSO-*d*_6_, ppm): 19.8 (2), 86.9, 114.2 (2), 127.4, 128.6 (2), 134.1, 145.3, 162.0 (2), 173.2, 209.0 (C=S). Anal. Calcd for C_14_H_16_N_6_O_2_S_2_ (364): C, 46.14; H, 4.43; N, 23.06. Found: C, 45.89; H, 4.78; N, 23.41.

#### 3.1.14. (*E*)-4-(5-Acetyl-4-hydroxy-2-(phenylimino)pyridine-1(2*H*)-yl)-*N*-cabamimidoylbenzenesul-fonamide (**15**)

A mixture of **5** (3.24 g, 0.01 mol), phenyl isothiocyanate (1.35 g, 0.01 mol) and sodium hydroxide (0.4 g, 0.01 mol) in ethanol (20 mL) was refluxed for 3 h. The reaction mixture was cooled, poured onto ice water, acidified with dilute HCl, then the solid product was filtered and crystallized from dioxane to give **15**. Yield%: 79, m.p. = 66.6 °C, IR, cm^−1^: 3436 (OH), 3208, 3186, 3121 (NH, NH_2_), 3030 (CH arom.), 2982, 2861 (CH aliph.), 1706 (C=O), 1596 (C=N), 1374, 1160 (SO_2_). ^1^H-NMR (DMSO-*d*_6_, ppm): 2.4[s, 3H, COCH_3_], 4.5[s, 1H, OH], 6.9[s, 2H, NH_2_, D_2_O-exchangeable], 7.1–7.4[m, 9H, Ar–H], 9.4[s, 1H, NH–imino, D_2_O-exchangeable], 10.8[s, 1H, SO_2_NH, D_2_O-exchangeable]. ^13^C-NMR (DMSO-*d*_6_, ppm): 26.2, 67.1, 118.1 (2), 121.6, 122.9 (2), 124.5, 128.6 (2), 128.9, 130.8 (2), 137.8, 142.4, 153.5, 164.9, 169.1, 171.4, 199.3. Anal. Calcd for C_20_H_19_N_5_O_4_S (425): C, 56.46; H, 4.50; N, 16.46. Found: C, 56.11; H, 4.89; N, 16.80.

### 3.2. In-Vitro Anticancer Screening

The human tumor cell line (MCF7) was available at the National Cancer Institute, Cairo, Egypt. The antitumor activity of the newly synthesized compounds was measured using the sulfo-rhodamine-B stain (SRB) assay by the method of Skehan *et al.* (1990) [35,36]. The cell lines were grown in RPMI 1640 medium containing 10% fetal bovine serum and 2 mM l-glutamine. Cells were plated in 96-multiwell plates (104 cells/well), after cell inoculation, the micro titer plates were incubated at 37 °C, 5% CO_2_, 95% air and 100% relative humidity for 24 h prior to addition of experimental drugs to allow attachment of cell to the wall of the plate. After 24 h, cell line was fixed *in situ* with TCA (trichloro acetic acid). Tested compounds **3**–**14** were dissolved in DMSO and diluted with saline to the appropriate volume and maintained in RPMI 1640 medium. Different concentrations of the compounds under test (5, 12.5, 25, and 50 μM) were added to the cell monolayer. Triplicate wells were prepared for each individual dose. Monolayer cells were incubated with the compounds for 48 h at 37 °C and in atmosphere of 5% CO_2_. After 48 h, cells were fixed *in situ* by the gentle addition of 50 μL of cold 30% (*w*/*v*) TCA (final concentration, 10% TCA) and incubated for 60 min at 4 °C. The supernatant was discarded; the plates were washed five times with tap water and air dried. Sulforhodamine B (SRB) solution (50 μL) at 0.4% (*w*/*v*) in 1% acetic acid was added to each of the wells, and plates were incubated for 20 min at room temperature. After staining, unbounded dye was removed by four washes with 1% acetic acid, and attached stain was recovered with Tris-EDTA buffer. Color intensity was measured in an ELISA reader (Gmbh, Viesbaden, Germany). The relation between surviving fraction and drug concentration was plotted to get the survival curve of each tumor cell line after the specified time. The concentration required for 50% inhibition of cell viability (*IC*_50_) was calculated and compared with the reference drug Doxorubicin and the results are given in (Table 1).

## 4. Conclusions

We report in this work the synthesis of a novel series of 4-(substituted)-*N*-(guanidinyl)benzenesulfonamides bearing a biologically active pyrazole, pyrimidine and pyridine moieties through simple and convenient routes These new derivatives were evaluated for their anticancer activity against human tumor breast cell line (MCF7). It was found that the most potent compounds in this study were the corresponding 2-cyanoacrylate **3**, 3-oxobutanoate **4**, pyrazole **6**, pyridine **9** and pyrazole **13**.

## Figures and Tables

**Table 1.** *In-vitro* anticancer screening of compounds **3**–**14** against human breast cell line (MCF7).

Compound No.	*IC*_50_ (μg/mL)	*IC*_50_ (μM)
**3**	16.7	49.5
**4**	19.4	54.8
**5**	24.4	75.3
**6**	19.0	59.0
**7**	23.6	70.2
**8**	29.0	68.1
**9**	21.7	59.2
**10**	23.3	76.1
**11**	24.2	75.6
**12**	34.4	102.9
**13**	22.9	57.8
**14**	NA	NA

Doxorubicin	39.0	71.8
